# Myocardial injury after endovascular revascularization in critical limb ischemia predicts 1-year mortality: a prospective observational cohort study

**DOI:** 10.1007/s00392-017-1185-z

**Published:** 2017-11-24

**Authors:** Wojciech Szczeklik, Marek Krzanowski, Paweł Maga, Łukasz Partyka, Jolanta Kościelniak, Paweł Kaczmarczyk, Mikołaj Maga, Patrycja Pieczka, Anna Suska, Agnieszka Wachsmann, Jacek Górka, Bruce Biccard, P. J. Devereaux

**Affiliations:** 1 Department of Intensive Care and Perioperative Medicine, Jagiellonian University Medical College, Krakow, Poland; 2 Department of Medicine, Jagiellonian University Medical College, ul. Skawińska 8, 31-066 Krakow, Poland; 3 Department of Angiology, Jagiellonian University Medical College, Krakow, Poland; 4 Department of Anaesthesia and Perioperative Medicine, Faculty of Health Sciences, University of Cape Town, Cape Town, South Africa; 5 Population Health Research Institute, McMaster University and Hamilton Health Sciences, Hamilton, ON Canada

**Keywords:** Endovascular treatment, Peripheral artery disease, Critical limb ischemia, Myocardial injury, Major adverse cardiovascular events

## Abstract

**Background:**

Patients with critical limb ischemia (CLI) are at increased risk of cardiovascular complications and mortality. To determine (1) incidence of myocardial injury following endovascular revascularization, and (2) relationship between myocardial injury with 1-year mortality and major adverse cardiovascular events (MACE; i.e., composite of myocardial infarction, stroke, and death).

**Methods and results:**

Single-center, prospective cohort study of CLI patients ≥ 45 years of age, who underwent endovascular revascularization with overnight hospitalization. High-sensitive troponins T (hsTnTs) were measured on admission, 3–6 h after endovascular revascularization and the subsequent morning. Myocardial injury after endovascular revascularization was defined as an hsTnT ≥ 14 ng/L with a relative increase ≥ 30% from the baseline value. We also evaluated other myocardial injury hsTnT thresholds (i.e., ≥ 30, ≥ 40, ≥ 60, and ≥ 80 ng/L). 239 consecutive patients (56% male, mean age 71.5 ± 10.1 years) were included; one patient was lost to follow-up. At 1 year, there were 34 deaths (14.2%), and 48 MACE (20.5%). Myocardial injury with the hsTnT threshold of 14 ng/L and relative increase by ≥ 30% from the baseline level occurred in 61 patients (25.5%). Myocardial injury was independently associated with 1-year mortality ([aHR], 2.44; 95% CI 1.18–5.06, for hsTnT ≥ 14 ng/L to aHR, 3.34; 95% CI 1.29–8.65 for hsTnT ≥ 80 ng/L). Myocardial injury was also independently associated with 1-year MACE ([AOR] 2.89; 95% CI 1.41–5.92 for hsTnT ≥ 14 ng/L to AOR, 6.69; 95% CI 2.17–20.68 for hsTnT ≥ 80 ng/L). 85.2% patients who had myocardial injury did not have ischemic clinical symptoms or electrocardiography changes. In sensitive analysis with exclusion of symptomatic patients that developed myocardial injury for the hsTnT ≥ 14 ng/L threshold, both the 1-year mortality (aHR: 2.19; CI 1.02–4.68; *p* = 0.04), and 1-year MACE (OR 2.25; CI 1.06–4.77; *p* = 0.036) remained significant.

**Conclusions:**

Myocardial injury is common following endovascular revascularization for CLI and associated with the risk of 1-year mortality and MACE.

## Introduction

Globally > 200 million individuals have lower extremity peripheral artery disease (PAD) [1–3]. The predominant etiology is atherosclerotic lesions that occlude the arterial lumen, compromising blood flow and resulting in ischemia. PAD shares several similarities in pathology and predisposing risk factors with coronary artery disease (CAD) and ischemic stroke [3, 4]. In all PAD patients, the risk of major adverse cardiovascular events (MACE) is three to sixfold higher than in the general population [5].

The most advanced stage of PAD is critical limb ischemia (CLI). This is a life-threatening condition [6], which affects approximately 1–2% of patients with PAD [2, 4]. The reported incidence of CLI ranges from 500 to 1000 per million annually in the general population, and has increased by over 20% in the past decade [6].

The standard treatment for CLI is revascularization, which should be attempted in the majority of patients without delay [6–8]. The aim of this treatment is to obtain straight line, unimpeded arterial flow to the foot in at least one vessel. This can be performed either with an open surgical or percutaneous endovascular approach [9]. Even with adequate treatment, CLI carries a substantial burden of disability, suboptimal quality of life, and substantial health care and social costs [6, 10]. Much of this is due to cardiovascular complications [11]. In the first year following the initial diagnosis of CLI mortality reaches 25% and surpasses 40% after 2 years [12–14]. In these cases, myocardial infarction is responsible for 40–60% of deaths and cerebral stroke for another 10–20% [5, 12].

One of possible explanations for this high risk of MACE is that revascularization procedures (both surgical and endovascular) may result in myocardial injury at the time of intervention that may impact future prognosis. This hypothesis is based on the data from the noncardiac surgical setting, where perioperative cardiac ischemia is common, and has been shown in large studies that postsurgical troponin elevation increases the risk of short and long-term mortality and MACE [15–19]. A new term—myocardial injury after noncardiac surgery (MINS) has been introduced in perioperative medicine [20], which carries a broader spectrum of myocardial injury than that defined by the universal myocardial infarction definition [21], and is frequent in vascular surgery [22].

Although MINS occurs after surgical revascularization for CLI and impacts outcomes, a little is known about the incidence and prognosis of myocardial injury following endovascular revascularization for CLI. Similar to surgery, endovascular revascularization may provoke myocardial ischemia, possibly by procedure-associated pain, catecholamine release, anemia, coagulation abnormalities, hypotension, tachycardia, and hypoxia [23, 24].

We conducted an observational cohort study in patients who underwent percutaneous endovascular revascularization for CLI conducted under local anesthesia to determine the incidence of myocardial injury and the relationship between myocardial injury and mortality and MACE at 1 year.

## Methods

### Study population

Between the years 2013 and 2015, we conducted a single-center, prospective, observational cohort study of CLI patients undergoing endovascular revascularization in the Vascular Diseases Department of the Jagiellonian University Hospital, Krakow, Poland. Eligible patients were ≥ 45 years of age and stayed at least overnight in hospital after the endovascular revascularization procedure. All patients fulfilled the definition of CLI according to current guidelines and were graded between 4 and 6 in the Rutherford classification [6, 10]. Patients were excluded from the study if they declined consent or if the baseline high-sensitivie troponin T (hsTnT) measurement or at least one post-procedural hsTnT measurement was not available.

The Jagiellonian University ethics committee approved the study. The protocol complied with the Helsinki Declaration, and all participants provided written informed consent before enrollment.

### Study procedures

#### Initial assessment

Research personnel interviewed patients and reviewed their medical records to obtain information on patients’ medical history. Before the endovascular revascularization all patients had an ECG performed and blood samples were taken at admission for C-reactive protein (CRP) and cardiac biomarkers [i.e., N-terminal brain-type natriuretic peptide [NT-proBNP] and hsTnT (both Elecsys 2010 analyzer; Roche)]. Additional hsTnT measurements were performed 3–6 h after endovascular revascularization and the following morning. The 99% threshold for hsTnT in healthy population is 14 ng/L (coefficient of variations < 10%) [25]. Preoperative estimated glomerular filtration rate (eGFR) was calculated using CKD-Epi equation and latest available preoperative serum creatinine value. Patients with an hsTnT ≥ 14 ng/L after their procedure had an ECG performed and the presence of any clinical ischemic symptoms were recorded (e.g., chest pain, dyspnea).

#### Follow-up

Research personnel followed patients throughout their hospitalization, and follow-up visits occurred in outpatient clinic at 1 month, 6 months and 1 year after endovascular revascularization. If the patient died or suffered MACE, the research team obtained the source documentation. Patients who either refused or were not able to come for the follow-up visits were contacted by phone, or their next-of-kin were contacted if study personnel could not reach the patient.

#### Endovascular revascularization

Endovascular revascularization was performed under local anesthesia. Once vascular access was established, the obstructive lesions were crossed by means of various catheters and guidewires. Next, the lumen was dilated, and either isolated (in 74 patients, 31%) percutaneous transluminal angioplasty (PTA) or PTA with subsequent bare metal stent implantation (in 165 patients, 69%) was performed. No drug-coated balloons were used in this study. All intravascular instrumentations were performed under angiographic guidance. At the end of the procedure, the vascular sheath was removed and the puncture site was sealed by manual compression or using closure devices.

### Study outcome measurements

We defined myocardial injury following endovascular treatment as:

1. Post-procedural hsTnT ≥ 14 ng/L

with

2) ≥ 30% relative increase from the baseline hsTnT measurement.

We also assessed other pre-specified myocardial injury thresholds: hsTnT ≥ 30; ≥ 40; ≥ 60; and ≥ 80 ng/L with the same obligatory relative increase of at least 30% from the baseline hsTnT level.

As there were no existing definitions of post-procedural myocardial injury at the time of the study commencement, the chosen obligatory relative increase of 30% in the hsTnT was based on previous studies that evaluated the delta changes in high-sensitive troponins levels, and suggested improved specificity for myocardial ischemia for this particular threshold [26].

The primary outcome was 1-year mortality, defined as death from any cause. The secondary outcome was major adverse cardiovascular event (MACE) that was defined as a composite of myocardial infarction, stroke and death (by any cause). If two events occurred in one patient (e.g., myocardial infarction followed by death) the patient was counted as having a single MACE. All potential MACE events were adjudicated independently by two physicians (internist and cardiologist).

### Statistical analyses

Categorical variables are presented as counts (percentages), whereas continuous variables are reported as medians (25–75 quartile range) unless otherwise specified.

Categorical variables were compared between the groups who did and did not have myocardial injury by the chi-square or the Fisher’s exact tests, and continuous variables were compared using the Student’s *t* test or the Mann–Whitney *U* test as appropriate.

We undertook multivariable regression analyses and assessed the relationship between different thresholds of myocardial injury that occurred after revascularization with the outcomes, specifically the Cox proportional hazard regression for evaluation of the mortality and logistic regression analysis for MACE. The independent variables included in the models by forced simultaneous entry were sex, Rutherford grade, CAD, history of MI, diabetes mellitus, and preoperative levels of eGFR, NT-proBNP, and hsTnT.

We reported results as hazard ratios (HR); for Cox analyses and odds ratios (OR) for logistic regression, with the corresponding 95% confidence intervals and associated *p* values. As a measurement of discrimination, we assessed the C-index for Cox analyses and area under the curve (AUC) for logistic regression analyses. For all tests, we used a two-sided alpha < 0.05 level of significance. All statistical analyses were performed using R software, version 3.3.2 (Vienna, Austria).

## Results

### Patients and preoperative cardiac biomarkers

**Fig. 1:**
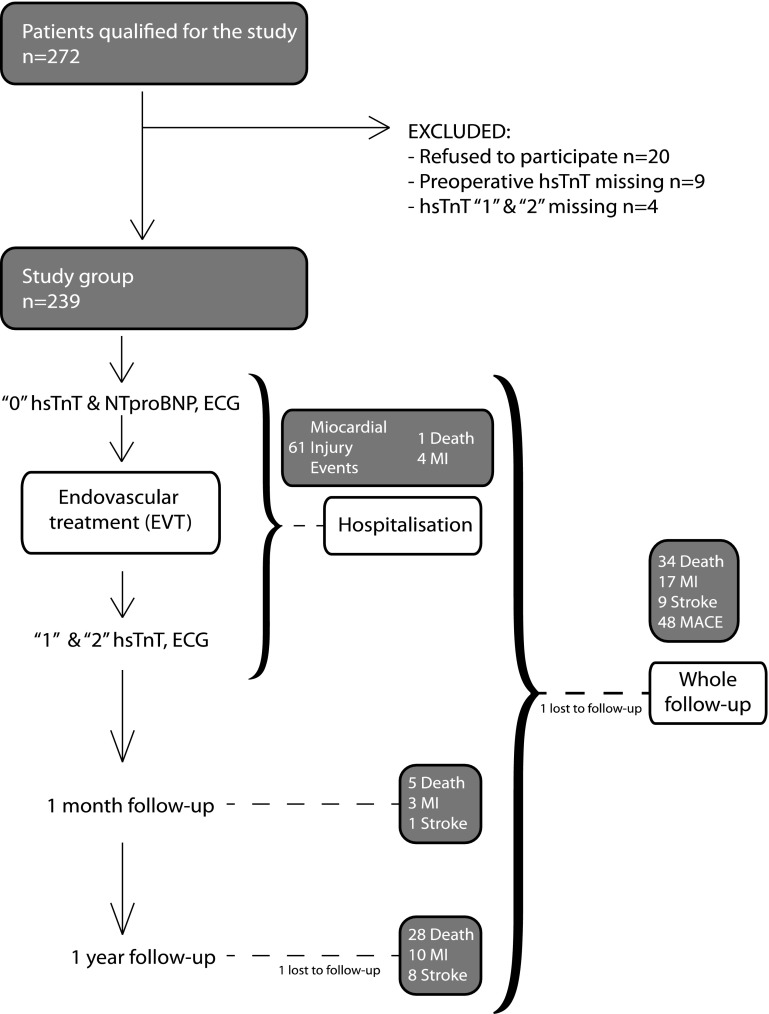
Study flow chart

Two hundred and thirty-nine consecutive patients were included; all patients were Caucasian, 56% were male, and the mean age was 71.5 ± 10.1 years. Almost 80% of patients had a Rutherford grade ≥ 5 before their procedure, and most had multiple risk factors for cardiovascular diseases (e.g., 48% had known coronary artery disease and 20% congestive heart failure). Only one patient was lost to follow-up at 1-year. Figure 1 presents the study flow chart, and Table 1 reports the clinical and laboratory characteristics of included patients. During the 1-year follow-up period, MACE occurred in 48 patients (20.5%) and 34 patients died (14.2%). Outcomes up to 1-year are presented in Table 2.

**Table 1:** Baseline characteristics

	All patients (*n* = 239)	Patients without myocardial injury (*n* = 178)	Patients with myocardial injury (*n* = 61)	*p* value
Age (years) (mean ± SD)	71.5 ± 10.1	70.5 ± 10.2	74.4 ± 9.4	0.007
Age > 75 years *n* (%)	97 (40.6%)	62 (34.8%)	35 (57.3%)	0.003
Male *n* (%)	134 (56.1%)	106 (59.6%)	28 (45.9%)	0.07
BMI kg/m^2^ (mean ± SD)	26.8 ± 4.7	26.8 ± 4.6	27.1 ± 5.3	0.809
Past medical history				
Hypertension *n* (%)	183 (76.6%)	133 (74.7%)	50 (82%)	0.295
Diabetes mellitus *n* (%)	138 (57.7%)	100 (56.2%)	38 (62.3%)	0.454
Insulin treatment	108 (45.2%)	78 (43.8%)	30 (49.2%)	0.551
CAD *n* (%)	115 (48.1%)	81 (45.5%)	34 (55.7)	0.184
Myocardial infarction *n* (%)	59 (24.7%)	41 (23.0%)	18 (29.5%)	0.308
TIA/stroke *n* (%)	44 (18.4%)	32 (18.0%)	12 (20%)	0.848
Chronic heart failure *n* (%)	48 (20.1%)	28 (15.7%)	20 (32.8%)	0.006
Dialysis *n* (%)	12 (5.0%)	8 (4.5%)	4 (6.6%)	0.508
Prior smoker *n* (%)	170 (71.1%)	126 (70.8%)	44 (72.1%)	0.872
Current smoker *n* (%)	30 (12.6%)	25 (14.0%)	5 (8.2%)	0.271
Rutherford 4 *n* (%)	49 (20.5%)	40 (22.5%)	9 (14.8%)	0.2698
Rutherford 5 *n* (%)	150 (62.8%)	112 (62.9%)	38 (62.3%)	1
Rutherford 6 *n* (%)	40 (16.7%)	26 (14.6%)	14 (23%)	0.163
Treatment				
ASA	234 (97.9%)	174 (97.8%)	60 (98.4%)	1
Clopidogrel	227 (95.0%)	171 (96.1%)	56 (91.8%)	0.1895
Statin	208 (87.0%)	160 (89.9%)	48 (78.7%)	0.044
B-blocker	141 (59.0%)	95 (53.4%)	46 (75.4%)	0.003
ACE inhibitor	144 (60.3%)	104 (58.4%)	40 (65.6%)	0.365
Heparin	165 (69.0%)	118 (66.3%)	47 (77%)	0.149
Oral diabetes drugs	55 (23.0%)	42 (23.6%)	13 (21.3%)	0.86
Laboratory parameters				
Hb g/dL	12.4 (10.9–13.5)	12.6 (11.3–13.6)	11.4 (10.6–12.4)	0.001
WBC count × 10^3^ cells/μ/L	8.2 (6.9–10.8)	8.0 (6.74–10.8)	8.7 (7.5–10.9)	0.04
Platelet count × 10^3^ cells/μ/L	233.5 (190–311)	230 (190–307)	255 (194–319)	0.622
CRP mg/L	7.4 (2.1–41.2)	6.1 (1.8–28.8)	15.5 (2.8–61.2)	0.019
CRP > 5 mg/L *n* (%)	144 (60.3%)	100 (56.2%)	44 (72.1%)	0.034
Creatinine μmol/L	83 (67–102)	81 (65.5–99)	87 (68.8–110)	0.22
eGFR	74 (56–96)	78 (57.3–98.8)	65.5 (50.3–87.8)	0.031
Preoperative hsTnT ng/L	18 (12–29)	17 (11–27)	22 (14–35.5)	0.017
NT-proBNP pg/ml median (Q1–Q3)	558 (195–1565)	459 (159–1365)	1061 (471–2490)	< 0.001

Continuous variables expressed as medians with 25–75 quartile range unless otherwise specified

*ACE* angiotensin converting enzyme, *ASA* acetylsalicylic acid, *BMI* body mass index, *CAD* coronary artery disease, *CRP* C-reactive protein, *GFR* glomerular filtration rate, *HgB* hemoglobin, *hsTnT* high-sensitive troponin T, *NT-proBNP* N-terminal brain natriuretic peptide, *SD* standard deviation, *TIA* transient ischemic attack, *WBC* white blood cells

**Table 2:** Outcomes in studied group

	All patients (*n* = 239)	Patients without myocardial injury (*n* = 178)	Patients with myocardial injury (*n* = 61)	*p* value
Death (up to 1 year) *n*%	34 (14.2%)	19 (10.7%)	15 (24.6%)	0.011
MI (up to 1 year) *n*%	16 (6.7%)	7 (3.9%)	9 (14.8%)	0.007
Stroke (up to 1 year) *n*%	9 (3.8%)	6 (3.3%)	3 (4.9%)	0.697
Composite end point (up to 1 year) *n*%	48 (20.1%)	27 (15.2%)	21 (34.4%)	0.003
Amputation below knee (up to 1 year) *n*%	8 (3.3%)	6 (3.4%)	2 (3.2%)	1
Amputation above knee (up to 1 year) *n*%	23 (9.6%)	18 (10.1%)	5 (8.2%)	0.804
Re-intervention (up to 1 year) *n*%	106 (44.3%)	83 (46.6%)	23 (37.7%)	0.237

Continuous variables expressed as medians with 25–75 quartile range unless otherwise specified

*MI* myocardial infarction

In 232 patients (97.1%), hsTnT in all three points of measurement were available for the analysis (seven patients did not have hsTnT measured on the morning following endovascular revascularization). Median baseline hsTnT level was 18 (12–29) ng/L and 151 patients (62.1%) had an hsTnT above the threshold level of 14 ng/L. Median baseline NT-proBNP was 558 (195–1565) pg/mL (Table 2), and in 78 patients (32%) the level of NT-proBNP was above the age-adjusted reference value [27].

### Myocardial injury after endovascular revascularization

Median hsTnT was 18 (12–31.5) after 3–6 h from treatment and 21 (13–41) on the following morning. Myocardial injury based on the hsTnT threshold ≥ 14 ng/L and relative troponin increase of at least 30% occurred in 61 patients (25.5%). Patients with myocardial injury (≥ 14 ng/L) when compared to those without myocardial injury, were older, more frequently had congestive heart failure, beta-blocker usage, and higher baseline levels of white blood cells, CRP, baseline hsTnT and NT-proBNP and lower baseline levels of eGFR and hemoglobin. The comparison of patients’ characteristics, treatments and laboratory findings between those that suffered myocardial injury (≥ 14 ng/L) and that did not is presented in Table 1.

In the myocardial injury (≥ 14 ng/L) group, the peak (maximum) hsTnT was found in 16.3% of patients 6–12 h after the revascularization, and in the majority (83.7%) by the next morning. Only six patients (9.8%) with myocardial injury (≥ 14 ng/L) had ischemic symptoms (e.g., chest discomfort was present in three) and presumed new ECG ischemic changes. Three additional patients had new ECG ischemic finding without clinical symptoms. In total, nine patients (14.8%) in the myocardial injury group presented with any ischemic symptoms (clinical symptoms and/or ECG changes).

### Myocardial injury and 1-year outcomes

Patients who developed myocardial injury were more likely to die or suffer MACE within the 1-year follow-up after revascularization compared to the patients who did not have myocardial injury. 17/34 (50%) of patients that died in the first year had myocardial injury (≥ 14 ng/L). The outcomes in unadjusted analysis for both 1-year mortality and MACE for different hsTnT thresholds for myocardial injury are presented in Table 3.

**Table 3:** Univariate comparison of patients that suffered myocardial injury (with different hsTnT thresholds) and those that did not in terms of 1-year outcomes

Myocardial injury TnT threshold (ng/L)	Presence of myocardial injury*	ALL *n* = 239	Death at 1-year	OR (95%CI) unadjusted	*p* value	MACE	OR (95%CI) unadjusted	*p* value
14	No	178 (74.5%)	19 (10.7%)	Reference		27 (15.1%)	Reference	
	Yes	61 (25.5%)	15 (24.6%)	2.73 (1.3–5.8)	0.011	21 (34.4%)	2.94 (1.50–5.73)	0.003
30	No	197 (82.4%)	23 (11.7%)	Reference		33 (16.8%)	Reference	
	Yes	42 (17.6%)	11 (26.2%)	2.68 (1.19–6.06)	0.026	15 (35.7%)	2.76 (1.33–5.75)	0.01
40	No	204 (85.4%)	23 (11.3%)	Reference		34 (16.7%)	Reference	
	Yes	35 (14.6%)	11 (31.43%)	3.61 (1.56–8.32)	0.004	14 (40%)	3.33 (1.54–7.20)	0.003
60	No	216 (90.4%)	25 (11.6%)	Reference		37 (17.1%)	Reference	
	Yes	23 (9.6%)	9 (39.1%)	4.91 (1.93–12.5)	0.0016	11 (47.8%)	4.43 (1.82–10.8)	0.002
80	No	222 (92.9%)	27 (12.2%)	Reference		38 (17.1%)	Reference	
	Yes	17 (7.1%)	7 (41.2%)	5.06 (1.78–14.4)	0.004	10 (58.8%)	6.92 (2.48–19.3)	< 0.001

*Myocardial injury for different post-procedural hsTnT thresholds with obligatory relative increase ≥ 30% from the baseline hsTnT measurement

*CI* confidence interval, *MACE* major adverse cardiovascular events, *OR* odds ratio

In the multivariable analysis, myocardial injury was found to be an independent predictor of both 1-year mortality and MACE. Adjusted hazard ratio for the association of myocardial injury after EVT with 1-year mortality varied from aHR: 2.44 (95% CI 1.18–5.01) for hsTnT ≥ 14 ng/L to aHR: 3.34 (95% CI 1.29–8.65) for myocardial injury after EVT ≥ 80 ng/L. Similar results were found for 1-year MACE with adjusted OR ranging from OR 2.89 (95% CI 1.41–5.92) to OR 6.69 (95% CI 2.17–20.68). Table 4 reports the 1-year mortality and MACE multivariable risk prediction models that included myocardial injury (≥ 14 ng/L). Table 5 reports the comparison of different myocardial injury thresholds on the risk of 1-year mortality and MACE.

**Table 4:** Multivariable risk prediction model of 1-year mortality and MACE in CLI patients including myocardial injury after endovascular revascularization (≥ 14 ng/L and ≥ 30% relative increase in hsTnT) and baseline cardiac biomarkers (hsTnT and NT-pro-BNP)

	1-year mortality		1-year MACE	
	Adjusted hazard ratio (95% CI)	*p* value	Adjusted OR (95% CI)	*p* value
Age	0.99 (0.95–1.033)	0.653	0.99 (0.96–1.03)	0.715
Sex				
Female	Reference		Reference	
Male	0.71 (0.31–1.64)	0.426	0.84 (0.39–1.82)	0.658
Rutherford				
4	Reference		Reference	
5	1.52 (0.50–4.59)	0.461	1.259 (0.49–3.26)	0.634
6	1.037 (0.26–4.17)	0.959	0.888 (0.25–3.10)	0.853
CAD	1.64 (0.66–4.09)	0.288	1.37 (0.56–3.35)	0.492
History of MI	0.87 (0.33–2.269)	0.768	1.19 (0.47–3.04)	0.714
DM	0.89 (0.41–1.93)	0.773	1.19 (0.57–2.48)	0.639
CRP	1.005 (0.999–1.011)	0.087	1.004 (0.997–1.01)	0.296
eGFR	1.002 (0.991–1.014)	0.719	0.9997 (0.9882–1.011)	0.956
NT-proBNP	1.00004 (0.999–1.00009)	0.149	1.00005 (0.99998–1.00012)	0.152
TnT0	1.002 (0.994–1.01)	0.649	0.999 (0.987–1.011)	0.868
Myocardial injury: hsTnT ≥ 14 ng/L with ≥ 30% relative hsTnT increase	2.44 (1.18–5.06)	0.016	2.89 (1.41–5.92)	0.004

CRP, eGFR, TnT and NT-proBNP expressed in the model as continuous variables. Cox proportional hazard analysis for the 1-year mortality model [C-index = 0.677 (SE = 0.051)] with the corresponding adjusted hazard radio (aHR) and 95% confidence interval (CI); multivariable logistic regression for 1-year MACE model [AUC = 0.65] with the corresponding adjusted odds ratio and 95% CI

*CAD* coronary artery disease, *CI* confidence interval, *CRP* C-reactive protein, *DM* diabetes mellitus, *eGFR* estimated glomerular filtration rate, *MACE* major adverse cardiovascular events, *MI* myocardial infarction, *NT-pro-BNP* N-terminal brain natriuretic peptide, *TnT* troponin T

**Table 5:** Summary of multivariable results for different hsTnT myocardial injury thresholds and risk prediction of 1-year mortality and MACE

Myocardial injury TnT threshold* (ng/L)	1-year mortality			1-year MACE		
	Adjusted hazard ratio (95% CI)	*p* value	C-index	Adjusted OR (95% CI)	*p* value	AUC
≥ 14	2.44 (1.18–5.06)	0.016	0.7	2.89 (1.41–5.92)	0.004	0.697
≥ 30	2.37 (1.09–5.17)	0.03	0.694	2.67 (1.20–5.90)	0.016	0.683
≥ 40	2.97 (1.35–6.55)	0.007	0.705	3.09 (1.33–7.14)	0.008	0.684
≥ 60	3.49 (1.49–8.14)	0.004	0.698	3.98 (1.50–10.56)	0.005	0.689
≥ 80	3.34 (1.29–8.65)	0.013	0.694	6.692 (2.17–20.68)	0.001	0.7

*Myocardial injury for different post-procedural hsTnT thresholds with obligatory relative increase ≥ 30% from the baseline hsTnT measurement

*AUC* area under the curve, *CI* confidence interval, *MACE* major adverse cardiovascular events, *OR* odds ratio

In sensitive analysis with exclusion of symptomatic patients that developed myocardial injury for the hsTnT ≥ 14 ng/L threshold (i.e., suffered MI), both the 1-year mortality (aHR: 2.19; CI 1.02–4.68; *p* = 0.04), and 1-year MACE (OR 2.25; CI 1.06–4.77; *p* = 0.036) remained significant.

## Discussion

Given the burden of cardiovascular complications following revascularization for CLI, there is a need to understand potential precipitants of these adverse outcomes. We evaluated the influence of myocardial injury after endovascular treatment in CLI patients on 1-year mortality and MACE. The findings show that (1) preoperative cardiac biomarkers (hsTnT and NT-proBNP) are frequently elevated in CLI patients, (2) almost 25% of CLI patients experience myocardial injury after endovascular treatment, and (3) myocardial injury is independently associated with 1-year mortality and MACE.

CAD is known to be present in 50–60% of patients with CLI [11, 28] and is included in all of the risk stratification tools as a predictor of mortality. In a recent study by Chen and colleagues, the presence of CAD in CLI patients undergoing diagnostic angiography or endovascular treatment was associated with higher rate of 5-year mortality and major cardiovascular complications [29].

However, the presence of CAD may be underestimated in patients with CLI, who often suffer from diabetes and immobilization and do not present with typical (angina) chest pain. A large trial that evaluated coronary angiograms before vascular surgery in almost 300 patients with CLI found that only 8% had coronary arteries without atherosclerosis [30].

The recent introduction of the hsTnT assay enables the detection of limited myocardial injury even without tissue necrosis, and together with BNP evaluation may help in diagnosing cardiac diseases. Both troponins and BNP have been broadly studied in several medical conditions and their elevation have been associated with the presence of coronary artery disease, congestive heart failure and overall poor prognosis [31–34].

Pohlhammer and colleagues demonstrated that hsTnT is elevated in males with PAD when compared to diabetes-matched controls, and that hsTnT elevation correlated with the presence of cardiovascular diseases [35]. In another study in CLI patients, elevated TnI was associated with increased mortality over a median of 8 months (HR 3.1; 95% CI 1.6–5.6) [36]. Both hsTnT [37] and BNP [38] among PAD patients were reported to be the highest in CLI patients.

In our study, we found that 62.1% of patients had pre-procedural hsTnT values above the 99% percentile of a normal reference level and 32% of patients had age-adjusted elevated NT-proBNP. According to past medical histories, the majority of patients had several cardiovascular comorbidities (e.g., hypertension, diabetes), with known coronary artery disease being present in 48% and known congestive heart failure in 20%. Considering the results of studied biomarkers cardiac disease may be underestimated in CLI patients.

Further, preoperative elevated cardiac biomarkers in CLI patients may reflect cardiac comorbidities (e.g., coronary artery disease, congestive heart failure and other cardiac conditions present in the clinical model), yet they may not be independently associated with post-procedural outcome (Table 4). Indeed, our data suggests that the only determinant of subsequent prognosis is dependent on monitoring the perioperative troponin response indicating myocardial injury.

The current study demonstrated a high prevalence of myocardial injury following revascularization. In the lowest evaluated threshold (hsTnT ≥ 14 ng/L; with at least 30% relative increase from the baseline level), myocardial injury was found in 25.5% of patients. Only a minority of patients (14.8%), who suffered myocardial injury after endovascular revascularization experienced clinical symptoms or had ECG findings indicative of myocardial ischemia. This would suggest that routine troponin monitoring is necessary in these patients, as opposed to symptom initiated troponin surveillance which would result in missing most of the patients who sustain myocardial injury. Despite being clinically silent, myocardial injury proved to be an independent predictor of both 1-year mortality and MACE. The incidence of 1-year mortality in the myocardial injury group was at least 25% in the lowest threshold and surpassed 40% in the highest. Similar findings were shown for 1-year MACE outcomes. These findings may partly explain why despite all the technical improvements in revascularization techniques, the mortality rate after revascularization has remained at a high level for the past several decades, [39] as we have not been able to adequately identify patients at risk without postoperative troponin surveillance.

To our knowledge, this is the first study evaluating myocardial injury after endovascular treatment in CLI patients with almost 100% complete follow-up.

Our study has limitations. Statistical association of myocardial injury with final outcomes was based on *a priori* specified thresholds. It is possible that our thresholds are not optimal, and should be re-evaluated in other studies. Troponins were measured after the treatment only at two time points with the possibility that we missed elevated troponins on the following days after EVT. Our approach to troponin surveillance was, however, pragmatic, as most of the patients are discharged on the day after endovascular revascularization, and further measurements would not be available. Finally, troponin elevation after endovascular revascularization might have resulted from etiologies other than ischemia. Further studies evaluating the mechanism of myocardial injury after endovascular revascularization are needed.

## Conclusions

Preoperative troponins and NT-proBNP are commonly elevated in patients with CLI undergoing endovascular procedures suggesting high risk of cardiac diseases. One in four revascularization patients experienced myocardial injury after endovascular revascularization with > 25% mortality within the subsequent year suggesting that myocardial injury is a trigger for future outcomes. The majority of patients who experienced myocardial injury after revascularization did not present with clinical signs or ECG changes indicative of myocardial ischemia, and would probably go unnoticed without troponin screening.
